# Eruptive Condyloma Accuminata after Initiation of Infliximab Treatment for Folliculitis Decalvans

**DOI:** 10.1155/2013/762035

**Published:** 2013-12-04

**Authors:** Douglas C. Wu, Thomas G. Salopek

**Affiliations:** ^1^Division of Dermatology, Department of Medicine, University of Alberta, Edmonton, AB, Canada T6G 2G3; ^2^Division of Dermatology and Cutaneous Sciences, Department of Medicine, University of Alberta, 2-125 Clinical Sciences Building, Edmonton, AB, Canada T6G 2G3

## Abstract

We report a patient with recalcitrant folliculitis decalvans who was placed on infliximab due to failure to respond to numerous immunosuppressive drugs and antibiotics. After the second infusion of infliximab the patient reported a cutaneous eruption to the bilateral groin, penis, scrotum, perineum, and perianal region consistent with genital warts. The case highlights the need to inquire about a past or current history of genital or anal warts prior to the initiation of anti-TNF therapy, particularly with infliximab. If present, consideration should be given to concurrent antiwart therapy.

## 1. Introduction

Folliculitis decalvans (FD) is classified as a neutrophilic primary cicatricial alopecia [1]. Clinically, FD usually affects the scalp and is characterized by erythematous follicular pustules and tufted folliculitis with multiple hairs emerging from a single dilated follicular ostia. Colonies of *Staphylococcus aureus* are often present [2]. FD is often recalcitrant to multimodal antimicrobial and anti-inflammatory therapy. Recently, Mihaljevic and Driesch reported the successful use of infliximab for the treatment of therapy-resistant FD [3]. Included in the potential risks of anti-TNF therapy is the increased susceptibility to *de novo* or reactivated infections. In this report, we detail the occurrence of eruptive condyloma accuminata after initiation of infliximab treatment for therapy-resistant folliculitis decalvans. This finding highlights the need to review with patients before initiating anti-TNF therapy the possibility of having warts of any kind and in particular genital warts.

## 2. Case Report

The case concerns a 47-year-old man with a ten-year history of recalcitrant FD. He initially presented with erythematous follicular pustules and scarring alopecia to the scalp. Incidentally, he was also noted to have findings consistent with a mild hidradenitis suppurativa (HS) to the groin. A biopsy of the scalp revealed very dense collections of inflammatory cells noted in the superficial reticular dermis and papillary dermis with numerous bacterial colonies within dilated follicular ostia. He underwent extensive treatment including courses of oral minocycline, clindamycin, rifampin, and dapsone; topical clindamycin and antiseptic shampoos; clobetasol 0.05% lotion and intralesional triamcinolone injections; acitretin and isotretinoin; prednisone, methotrexate, and mycophenolate mofetil. Unfortunately, these treatments and combinations thereof failed to produce a consistent remission and the patient remained on 15–20 mg daily of prednisone to achieve mediocre control of his condition.

**Figure 1:**
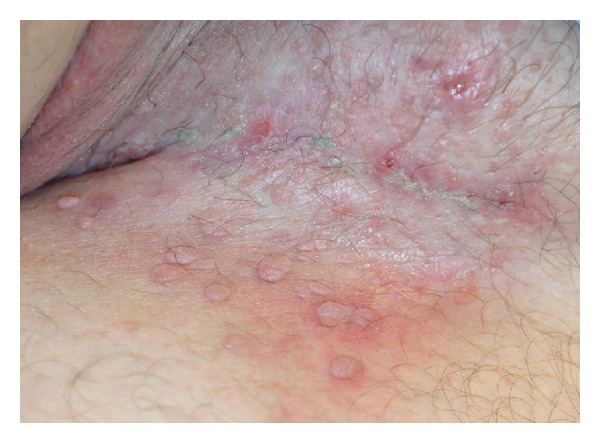
Multiple condyloma accuminata along the left inguinal fold.

In December 2012, infliximab infusions were initiated. Prior to commencement of infliximab therapy, it was ascertained that the patient was negative for HIV, hepatitis B, and hepatitis C. Additionally, a tuberculin skin test was measured at 0 mm and a screening chest X-ray revealed no evidence of tuberculosis. The patient received two infusions of infliximab at a dose of 5 mg/kg spaced 2 weeks apart. Shortly after his second infusion, the patient developed severe eruptive condyloma accuminata affecting his bilateral groin, penis, scrotum, perineum, and perianal region (Figure 1). Infliximab infusions were discontinued. His warts are presently being treated with a combination of cryotherapy, imiquimod 5% cream, and podofilox 0.5% solution. With application of this combination, the condyloma resolved rapidly.

## 3. Discussion

Reports of human papilloma virus (HPV) infection associated with anti-TNF therapy have thus far been rare [4–6]. Almost all cases have been associated with infliximab, although one patient developed perianal condylomata in association with etanercept. Other viral infections frequently seen by dermatologists that may be exacerbated by anti-TNF therapy include herpes zoster, primary varicella, and molluscum contagiosum [7, 8]. Our patient did not have a known previous history of genital condylomata and repeated clinical examinations of his groin region (for mild HS) did not identify any evidence of disease prior to infliximab infusions. It is possible that he was an asymptomatic carrier of HPV, which became clinically apparent only in the relatively immunosuppressed state of TNF inhibition. Of note, he was also on concurrent prednisone during his infliximab infusions and this may also have exacerbated the subsequent florid HPV eruption. Due to the risk of potential exacerbation of genital warts, patients should be informed and perhaps screened prior to initiation of anti-TNF therapy. If there is a history of verruca, consideration should be given to concurrent antiwart therapy.
